# A rare case of abdominal foreign bodies; laparoscopic removal of a sewing needle

**DOI:** 10.1016/j.amsu.2022.104747

**Published:** 2022-09-22

**Authors:** Ramin bozorgmehr, Mahsa Bahadorinia, Shiva Pouyanfar, Mojtaba ahmadinejad, Mohammad hadi bahri, Javad Zebarjadi Bagherpour

**Affiliations:** a Department of Surgery Shahid Madani Hospital, Alborz University of Medical Sciences Alborz, Iran; b Student Research Committee, Alborz University of Medical Sciences, Karaj, Iran

**Keywords:** Foreign body, Case report, Laparoscopy

## Abstract

**Background:**

Foreign body (FB) ingestion is a common condition. Mostly FBs are found ingested accidently or intentionally in children and adults with mental status alterations. Depending on the type of object, different complications can occur. There exist numerous methods for removing each specific FB. Fortunately, most FBs tend to move uneventfully through the gastrointestinal tract without any intervention; but managing some foreign objects can be difficult and lead to severe complications. Endoscopy helps with the diagnosis and treatment of these cases, but the time of the management plays an important role.

**Case presentation:**

A 26-year-old female who intentionally swallowed two sewing needles, presented to our emergency department with abdominal pain two months after the FB ingestion. One of the sewing needles was spontaneously excreted through the bowel, and the other was present in her body for two months. The FB had penetrated the stomach and migrated to the peritoneal cavity. The patient's condition was managed by laparoscopic removal of the FB and repair of the damaged tissue. According to the traumatic nature of the needle to abdominal viscera, and standing outside the GI tract on the pancreas surface, laparoscopic removal of the foreign metallic body was chosen to be performed. No complication was seen during the postoperative period.

**Conclusion:**

This report emphasizes the importance of prompt evaluation of FB patients and finding the appropriate method of managing its complications. Preventing complications requires focusing on symptoms and instant management of the ingested FBs.

## Highlights

• Foreign body (FB) ingestion is a common condition Depending on the type of object, different complications can occur.
• Managing these patients can be challenging due to the risk of damage that they may cause.
• Laparoscopy can be one of the suitable options with minimal tissue damage in the management of these patients.

## 1 Introduction

Ingested foreign bodies (FBs) can pass through the gastrointestinal (GI) tract, mostly without any problem and without intervention, but GI tract FBs can also become threatening depending upon the type of object thus carrying a higher risk of complications [1,2]. Although more common in children [2], a large variety of FBs in adults are found ingested accidently or intentionally, for example, in a state of mental condition [3]. Endoscopy prepares the ground for diagnosis and treatment of FB ingestions but to a large extent, the outcome depends on the time of management [4]. Furthermore, in the management of the FBs, some need an operative intervention due to complications or failure of other options [2]. FBs passing the GI tract may cause serious complications such as bowel obstruction, aspiration, hemorrhage, perforation, fistulization, abscess formation, septicemia, and death [2,5]. Here, we report a case of intentional ingestion of two sewing needles (2 months before admission) in a 26-year-old woman brought to the hospital with abdominal pain. FBs included two sewing needles, one of them found near the stomach which measured 5 cm in length and had perforated the stomach. The patient's condition was managed by laparoscopic removal of the FB and repair of the damaged tissue. Based on Surgical Case Report, 2020 (SCARE) guidelines [6].

## 2 Case presentation

A 26-year-old female was brought to our Emergency Department (ED) with abdominal pain and a history of intentional ingestion of two 5-cm-long sewing needles two months prior to presentation. Past medical and surgical history was nonspecific. The patient confirmed that one of the needles had been excreted in stool a few days after ingestion. The patient complained of epigastric pain starting two days earlier. The pain was positional (getting worse when bending forward), intermittently 2–3 times a day, each time lasting for about an hour, but was not related to oral intake of the regular diet. She denied any nausea or vomiting.

**Fig. 1:**
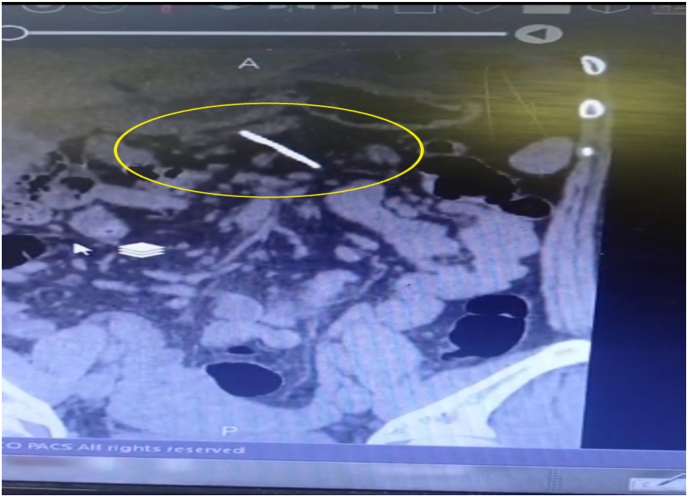
Coronal view,needle in surface of pancreas.

**Fig. 2:**
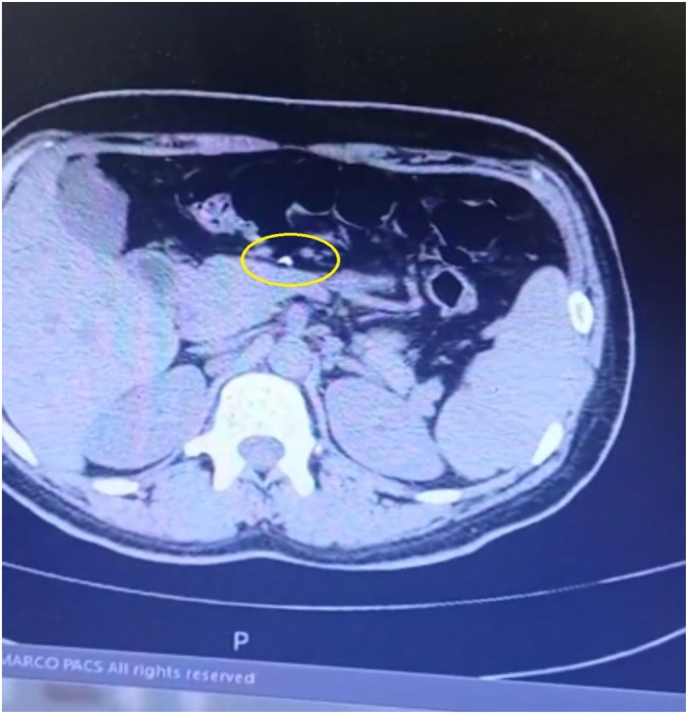
Axial view, needle in surface of pancreas.

**Fig. 3:**
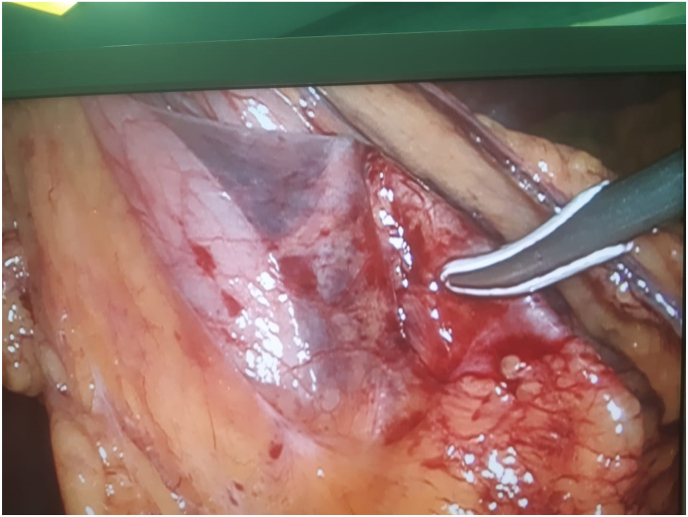
Intraoperative view of needle location.

**Fig. 4:**
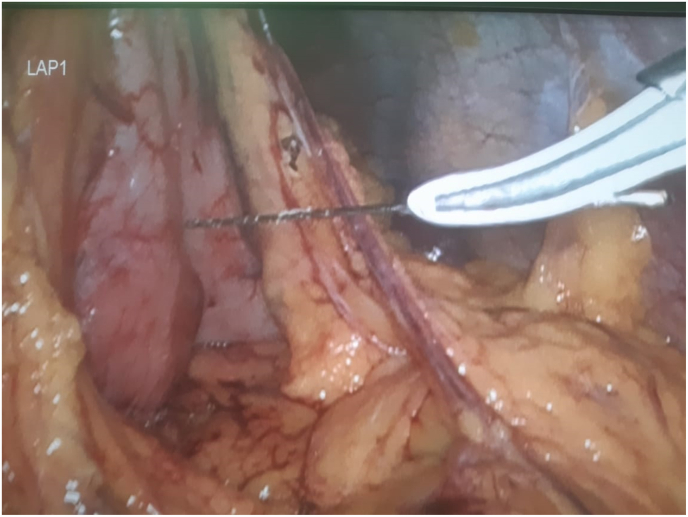
Removing needle from its location.

**Fig. 5:**
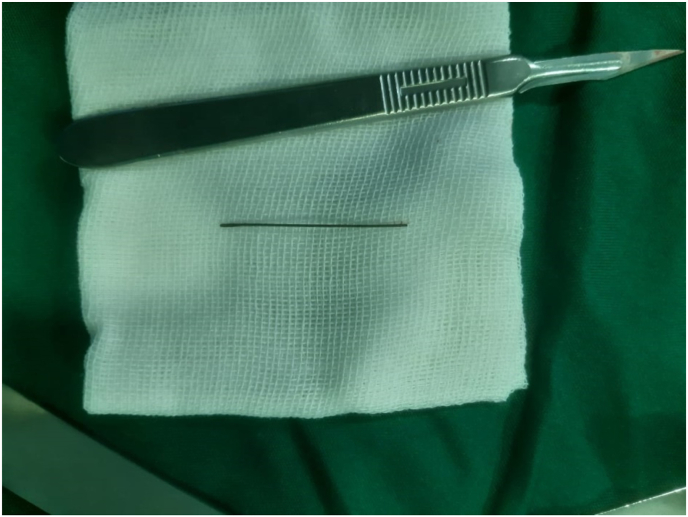
Needle after removal from abdomen.

On arrival, her general appearance was neither ill nor toxic, and she was completely alert and oriented with no acute distress. On physical examination, no oral lesion was seen. She had a temperature of 37.2 °C, a blood pressure of 100/70 mmHg, a respiratory rate of 17/min, and a pulse rate of 80/min. On heart auscultation, NL S1 S2 was noted. Lung auscultation appeared normal. The abdomen had normal bowel sounds and was soft, with localized tenderness in the epigastric region but without rebound tenderness or guarding. On rectal examination, no melena or stool was noted. No other notable abnormality was found on systemic examination. In blood tests, CBC differentials, liver function tests, pancreatic enzymes, and other parameters were within normal limits. The endoscopic evaluation confirmed that the esophagus, stomach, and duodenum had no apparent evidence of the presence of FB, perforation, or any other specific pathology. Computed tomography (CT) scan without contrast revealed the location of a needle outside the GI tract and on the pancreas surface (Fig. 1 coronal view and Fig. 2 axial view). According to the sharp and penetrative shape of the needle, and its traumatic nature to abdominal viscera, along with the fact that it was outside the GI tract, laparoscopic removal was performed by attending surgeon (Fig. 3: intraoperative view and Fig. 4 removing needle from its location). During the laparoscopic exploration, the gastrocolic ligament was opened with the help of a laparoscopic instrument. The foreign metallic body (sewing needle, Fig. 5) was seen on the pancreas surface and was removed gently and there was no organ damage of perforation during the operation. The patient spent the postoperative period in the general ward for two days. After one day, the patient was able to tolerate a semi-solid diet. No complication was seen during the 2-day hospital stay and postoperative period, and the patient was discharged home in good clinical condition and in 2 months after surgery there was no problem.

## 3 Discussion

FB ingestion is a common condition, and various objects could be found in different sites of the digestive tract, be it small or large, can become a threat to body organs. It can be strange as a corncob inserted into the rectum, or ingested dental bridges, cocaine packs, bones, and different metallic objects such as needles, pins, or coins. Most FBs find their way out of the body spontaneously, but in a small number of patients, getting the FB out of the GI tract carries some problems and causes complications due to the trauma and obstruction that has been set up. Ingested FBs usually don't migrate from the GI tract to the abdominal viscera, but rarely, as in our case, the sharp-pointed object penetrates through the wall of the GI tract into the pancreas or liver [7,8]; considering certain loop-like areas in the GI tract being ideal for this to happen. Patients may show little or no symptoms for a long time and may not seek medical help on time, which leads to late diagnosis and treatment and serious complications. Complications depend on the type of FB and its potential for perforation, obstruction, penetration, and causing a GI bleed. In patients who have ingested a FB and nothing is found in the GI tract on endo/colonoscopic examination, migration out of the GI tract should be kept in mind, especially if the FB has a penetrating nature. Further, this migration can cause pancreatitis and in the long term, abscess formation [8], mimicking symptoms of other diseases [9]. These conditions should be managed as conservatively as possible; fortunately, surgical procedures are the last and least method to remove the FBs, and laparoscopic removal is the first choice in this area [9], but the longer we wait, the more probability of choosing the open method over laparoscopic approach. Intentionally ingested FBs are mainly sharp metallic objects, and impaction duration is longer; also, the endoscopic treatment success rate is lower than that of the general population [3].

We, in this article, reported the case of a 26-year-old female who presented to the hospital two months after she swallowed two metallic needles intentionally. She was stable at the presence to the hospital and complained of abdominal pain. After evaluating the condition, we prepared for the laparoscopic removal of the FB for it offered advantages of surgery without the incision of laparotomy. However, the two-months delay in the management of this condition caused the penetration of a needle onto the surface of the pancreas.

Imaging modalities can help us detect the presence and location of FB and provide the basis for our management; although, we should be careful of suspected metallic FBs in patients who are undergoing a magnetic resonance imaging study. When FB presence is suspected, the clinician needs to take a thorough medical history in order to prevent unwanted events and also for appropriate management of the patient.

Choosing the right treatment method is obliging and can be done according to the shape, location, nature of the FBs, and patient stability. The presence of such a sharp-pointed foreign object lodged inside the body for weeks should be considered a serious condition even with no alerting symptoms.

## 4 Conclusion

We reported an uncommon case of intentional ingestion of sharp metallic FBs, trapped in the abdominal viscera for two months. Managing these patients can be challenging due to the risk of damage that they may cause. Prompt evaluation of the patients and choosing the right treatment method are obliging. Attention should be focused on symptoms and management of the ingested FBs to prevent complications.

## Funding

The author(s) received no financial support for the research, authorship, and/or publication of this article

## Sources of funding

There in no funding sources.

## Ethical approval

This article is a case report and all patient informations is secret.

## Consent

Informed consent was obtained from patient, for publication of this case report and accompanying images. A copy of the written consent is available for review by the Editor-in-Chief of this journal on request.

## Author contribution

All authores has same contribution in this article.

## Registration of research studies

1. Name of the registry:
2. Unique identifying number or registration ID:
3. Hyperlink to your specific registration (must be publicly accessible and will be checked):

## Guarantor

Dr Javad zebarjadi accepts full responsibility for the work and/or the conduct of the study, had access to the data, and controlled the decision to publish.

## Provenance and peer review

Not commissioned, externally peer-reviewed.

## Declaration of competing interest

All authors confirm that there is no conflict of interest.
